# Dissolution Enhancement in Cocoa Extract, Combining Hydrophilic Polymers through Hot-Melt Extrusion

**DOI:** 10.3390/pharmaceutics10030135

**Published:** 2018-08-21

**Authors:** Ludmila A. G. Pinho, Saulo G. Souza, Ricardo N. Marreto, Livia L. Sa-Barreto, Tais Gratieri, Guilherme M. Gelfuso, Marcilio Cunha-Filho

**Affiliations:** 1 Laboratory of Food, Drugs and Cosmetics (LTMAC), University of Brasília (UnB), 70910-900 Brasília, DF, Brazil; ludmila.alvim@gmail.com (L.A.G.P.); godoysags@gmail.com (S.G.S.); liviabarreto@unb.br (L.L.S.-B.); tgratieri@gmail.com (T.G.); gmgelfuso@unb.br (G.M.G.); 2 School of Pharmacy, Federal University of Goiás, 74 605-170 Goiânia, GO, Brazil; rnmarreto@gmail.com

**Keywords:** hot-melt extrusion, cocoa extract, mixture design, flowability, dissolution rate

## Abstract

The aim of this study was to improve the physicochemical properties of cocoa extract (CE) using hot-melt extrusion (HME) for pharmaceutical proposes. A mixture design was applied using three distinct hydrophilic polymeric matrices (Soluplus, Plasdone S630, and Eudragit E). Systems obtained by HME were evaluated using morphologic, chromatographic, thermic, spectroscopic, and diffractometric assays. The flow, wettability, and dissolution rate of HME powders were also assessed. Both CE and its marker theobromine proved to be stable under heating according to thermal analysis and Arrhenius plot under isothermal conditions. Physicochemical analysis confirmed the stability of CE HME preparations and provided evidence of drug–polymer interactions. Improvements in the functional characteristics of CE were observed after the extrusion process, particularly in dissolution and flow properties. In addition, the use of a mixture design allowed the identification of synergic effects by excipient combination. The optimized combination of polymers obtained considering four different aspects showed that a mixture of the Soluplus, Plasdone S630, and Eudragit E in equal proportions produced the best results (flowability index 88%; contact angle 47°; dispersibility 7.5%; and dissolution efficiency 87%), therefore making the pharmaceutical use of CE more feasible.

## 1. Introduction

Cocoa extract (CE; *Theobroma cacao* L.), one of the main Brazilian agricultural commodities, is largely composed of the flavonoid theobromine (TB) [1]. Besides its common use in the food industry, this natural product has exhibited diverse therapeutic potential, such as cardioprotective and anti-inflammatory actions [1]. Also, TB has shown a variety of possible pharmacological applications, including use as anti-carcinogenic or anticholesterolemic agents, and as a cough suppressant [2,3,4].

Vegetal sources have been widely explored therapeutically by traditional medicine; however, the insertion of new technologies in the development of this class of products is quite scarce, maintaining its “homemade” character. Furthermore, the poor mechanical and physicochemical properties of dried vegetal extracts can result in deficient flow behavior and inadequate compactness, hindering large-scale production [5]. Indeed, CE has remarkably deficient flow and wettability characteristics [6], while TB has restricted solubility and a poor dissolution profile [7,8].

Hot-melt extrusion (HME) has gained interest in the pharmaceutical field as a processing technology capable of producing solid dispersions with a high degree of drug-polymer interactions. The simultaneous mechanical and thermal shear of samples achieved by HME can noticeably modify drug properties such as solubility [9], poor taste, and flowability [10], while producing sustained drug delivery systems [11]. Moreover, the association of HME with computer-aided design and computer-aided manufacturing is expanding its medical potential in the production of prostheses and even in 3D printed drug products [12,13]. Meanwhile, the use of this technology surpasses the limitations of more traditional methods that also produce drug solid dispersions, such as lyophilization or spray-drying. Samples are rapidly processed by HME in a continuous single step without requiring the use of organic solvents, and production is easily scaled up [14].

Despite such advantages, the use of HME for treating vegetal products is still insufficiently explored. The literature has described only a few recent studies using curcumin [15], the herb *Angelica gigas* Nakai [16], and *Ginkgo biloba* [17]. All developed formulations have shown encouraging results for obtaining novel natural pharmaceutical products.

In view of this background, this paper evaluated the feasibility of producing solid CE dispersions by means of HME and the resulting solid dispersions’ enhanced flowability and solubility. In this way, a mixture design was used to find an optimized formulation composition that could improve the pharmaceutical properties of CE. Three distinct hydrophilic polymeric matrices commonly used in HME were tested, namely Soluplus (Sol), Eudragit E (EuE), and Plasdone S (PVP).

## 2. Materials and Methods

### 2.1. Materials

Powdered CE containing 20% of TB (lot CAC01) was obtained from Badmonkeys Botanicals (Tacoma, WA, USA). TB (lot BCBM9560V, >98.5%) was purchased from Sigma-Aldrich (Steinheim, Germany). The polymer Plasdone^®^ S-630 (lot 0001810863, poly(vinylpyrrolidone-*co*-vinylacetate); PVP) was kindly provided by Ashland Specialty Ingredients (Covington, LA, USA); Soluplus^®^ (lot 844143368EO, polyvinylcaprolactam-polyvinylacetate-polyethyleneglycol, Sol) was donated by BASF (Ludwigshafen, Germany); and Eudragit^®^ E PO (lot G130531504, butyl methacrylate: dimethylamino ethyl methacrylate: methyl methacrylate 1:2:1, EuE) was donated by the Evonik Corporation (Essen, Germany). All other chemicals and solvents were of analytical grade.

### 2.2. Mixture Design

A simplex centroid mixture design with three components without constraints was used in order to determine the ideal combination of excipients to improve CE properties (Table 1) [14]. The responses obtained for the flowability, dispersibility, contact angle and dissolution rate were analyzed using the software Design Expert 8.0 (Stat-Ease, Minneapolis, MN, USA). The possible mathematic models were examined using one-way ANOVA. The best-fitting model was selected for each response based on *F*-values and *p*-values, and the predictive equations containing only significant terms were built from stepwise multiple regression analysis.

The optimized response was calculated from the all responses obtained, considering the formulation conditions for maximum dissolution and flowability with an importance score value of 3 since those are assays directly accessed by the pharmaceutical industry, and minimizing the dispersibility and contact angle with an importance score value of 1 since they are complementary assays.

### 2.3. Hot-melt extrusion (HME) Preparation

Physical mixtures (PMs) of CE and the selected polymers (PVP, Sol, or EuE) were prepared with a mortar and pestle, maintaining the CE–polymer proportion of 3:7 (*w*/*w*). The proportion was chosen based on previous tests. The PMs were used to manually feed a co-rotating conical twin-screw extruder (HAAKE MiniCTW, ThermoScientific, Waltham, MA, USA). Material characteristics were exploited to set the extrusion conditions, which were adjusted to allow the appropriate extrusion of each material with continuous flow. The temperature and rotation used are described in Table 1. After extrusion, formulations were milled in a knife mill to meet the particle size range of 125–250 µm.

### 2.4. Drug Determination

Quantification of TB within CE, in extrudates, and in dissolution studies was performed through a reversed-phase chromatographic method with UV detection at 274 nm using the HPLC model LC-20AT (Shimadzu, Kyoto, Japan). The method was adapted from a previous report [18]. The operating conditions of the method were as follows: 10 μL of injection volume; reversed-phase C18 column (LC Column, 300 × 3.9 mm, 10 μm); phosphoric acid 0.01 mol L^−1^/methanol (75:25, *v*/*v*) as mobile phase; and flow rate of 1.2 mL min^−1^. The method was validated following the International Conference on Harmonization parameters and proved to be selective against the polymers and CE interferents and linear (*r* = 0.9997). The limits of quantification and detection were 0.15 μg mL^−1^ and 0.05 μg mL^−1^, respectively.

### 2.5. Morphological Analysis

The morphology of TB and CE, as well as of CE-polymer mixtures before and after HME processing, were assessed by optical microscopy using a stereoscope (Laborana/SZ; SZT, São Paulo, Brazil) coupled to a video camera and with the aid of a scanning electronic microscope (SEM; Jeol, JSM-7001F, Tokyo, Japan).

### 2.6. Thermogravimetric Analyses (TGA)

TGA were carried out using a DTG-60H (Shimadzu, Japan). Kinetic analyses using isothermal conditions were performed for TB and CE. Samples were heated at selected temperatures (chosen as those close to the initial temperature for each sample’s decomposition) and maintained at constant temperature until 5% of mass loss had occurred. An Arrhenius plot was built based on the experimental data, and the activation energy of each sample was calculated from the linear regression [19].

Linear heating experiments were performed in TB and CE, as well as in CE-polymer mixtures before and after HME processing using platinum pans and under a nitrogen atmosphere or synthetic air with a flow rate of 50 mL min^−1^ at a heating rate of 5 °C min^−1^ from 30 to 400 °C.

### 2.7. X-Ray Powder Diffraction (XRPD)

XRPD spectra of TB and CE as well as CE-polymer mixtures before and after HME processing were collected using a D8 FOCUS XRPD (Bruker, Billerica, MA, USA). The scan speed was 2 degrees min^−1^, and the step size was 0.02 degrees. The diffraction patterns were obtained at angles between 5 and 60° (θ–2θ).

### 2.8. Fourier Transform Infrared Spectroscopy (FTIR)

FTIR analyses of TB and CE as well as CE-polymer mixtures before and after HME processing were performed on a Varian 640 FTIR spectrometer using an ATR imaging accessory (Agilent Technologies, Santa Clara, CA, USA). Spectra were recorded between 4000 and 600 cm^−1^ at an optical resolution of 4 cm^−1^.

### 2.9. Flow Measurements

Multiple flow measurements were assessed using a powder characteristic tester, PT-N (Hosokawa Micron Powder Systems, Summit, NJ, USA). From these acquired data, powder flowability was determined according to Carr based on the angle of repose, angle of spatula, compressibility, and uniformity tests [20].

Dispersibility was calculated by dropping 10 g of the powder into a cylindrical tube up to a watch glass of 100 mm diameter positioned on the bottom. The amount of powder recovered from the watch glass was compared with the initial powder to calculate the dispersibility in percentages. All measurements were performed in triplicate, and the flowability index and dispersibility were calculated using the Hosokawa software.

### 2.10. Contact Angle Determination

The contact angle was measured by CAM-PLUS contact angle meter (ChemInstruments, Fairfield, OH, USA). Tablets of each system were prepared using a hydraulic press with a compression force of 4.28 kgf for 5 s. Purified water was dropped onto the surface of the tablets, for which the contact angles were immediately measured. Results are represented as the mean of 10 replicates.

### 2.11. Dissolution Studies

Dissolution profiles of CE and HME extrudates were determined in a dissolution system, Ethik model 299 (Nova Ética, São Paulo, Brazil), using 250 mL of HCl 0.1 mol L^−1^ as medium, following the FDA recommendations for immediate-release solid oral dosage forms. The temperature was maintained at 37 °C, and paddle speed was adjusted to 75 rpm. Samples containing the equivalent of 250 mg of TB, a possible therapeutic dose, were added to the dissolution vessel [8]. Aliquots of dissolution media were withdrawn at predetermined time intervals, filtered, and appropriated diluted for drug determination, as previously described. Experiments were performed in triplicate for each sample, and dissolution profiles were evaluated using their correspondent dissolution efficiency at 30 min (DE30). Results were graphically represented as the mean together with the DE30 data and its standard deviation [21,22].

### 2.12. Statistical Analysis

Statistical analysis was performed using GraphPad Prism 6 and IBM SPSS Statistics 22. Normality was previously tested for all data. Results with parametric behavior were compared using one-way ANOVA followed by Tukey post-test. Results with no parametric behavior were evaluated through Kruskal-Wallis test followed by Dunn’s post-test. Significance level (*p*) was fixed at 0.05. 

## 3. Results and Discussion

### 3.1. Thermal Stability of Theobromine (TB) and Cocoa extract (CE)

Thermogravimetric tests were performed in CE samples as well as in the main marker TB in order to evaluate thermal stability. TGA analysis of TB using a constant heating rate revealed a monophasic drug decomposition in the range of 230–330 °C (*T*_peak_ of first derivative = 305 °C), involving a complete sample weight loss. Decomposition of CE, meanwhile, occurred in a similar range of temperature (210–330 °C) as a well-defined decomposition first step with 43% weight loss (*T*_peak_ of first derivative = 279 °C), likely corresponding to the decomposition of TB and other compounds found in CE such as polyphenols and organic matter [23]. TGA performed in inert (N_2_) or oxidant atmosphere (synthetic air) showed the same profile, suggesting that oxygen did not affect the decomposition kinetics under the conditions studied.

The Arrhenius kinetic approach was applied based on TGA isothermal studies in an inert atmosphere. The activation energy values of TB and CE were 173.1 kJ mol^−1^ (*r* = 0.967) and 122.0 kJ mol^−1^ (*r* = 0.985), respectively, which are fairly high values when compared with those found for other drugs, which are usually within the range of 40–100 kJ mol^−1^ [24]. Indeed, TB activation energy is more than twice the value described for caffeine: 80.5 kJ mol^−1^ [25]. According to the Arrhenius plot, at the highest HME temperature used in this study (185 °C), TB and CE would take 163 min and 58 min, respectively, to lose 5% of their weight. Considering that the extrusion process takes less than 5 min, there are reasonable indications for the feasibility of thermal methods for CE processing without stability concerns.

### 3.2. Physicochemical Characterization

Extrusion conditions were settled to obtain continuous extrusion, and processing temperatures were established at the range of 150–185 °C, above at least 40 °C of the glass transition of polymers for single matrix systems based on previous studies with the same polymers [8]. In HME-Sol, the rotation speed and temperature were adjusted to have a uniform strip with better flow. For systems with more than one polymer, the initial settled temperature was the lowest one used for the present polymers, but it had to be increased for HME-Sol-EuE and HME-Sol-PVP to obtain a more homogeneous extrudate with adequate extrusion flow. The filaments showed a brown color and a uniform appearance (Table 1). HPLC analyses revealed no decomposition signal with drug content in the range of 90–104%.

**Figure 1:**
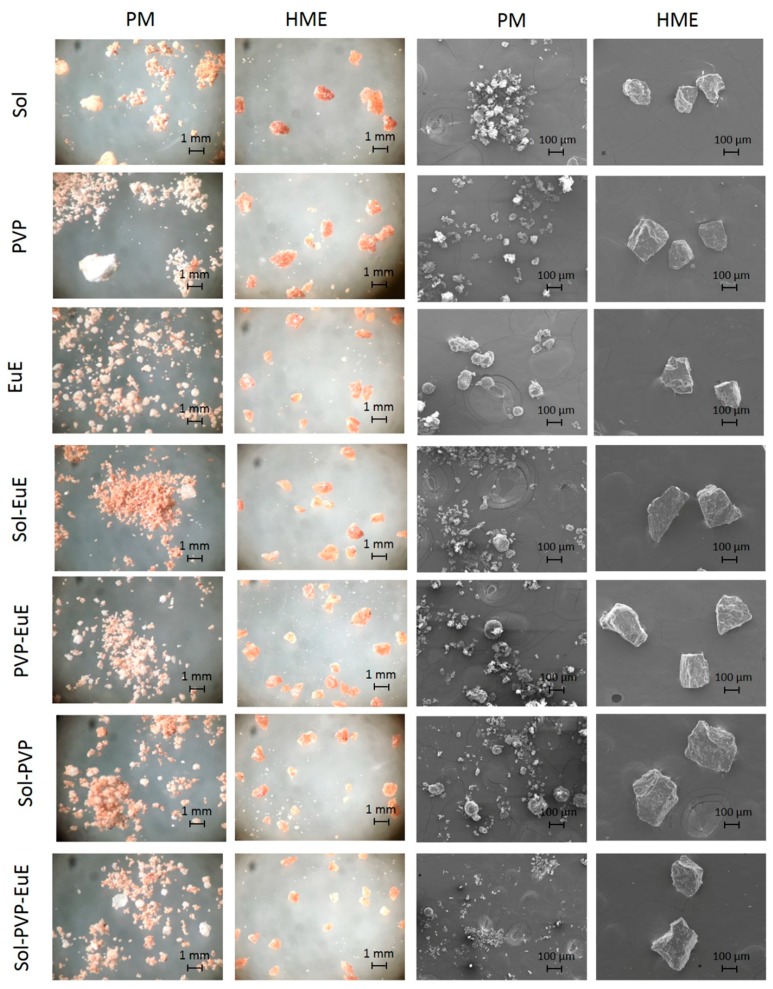
Optical and scanning electronic microscope (SEM) micrographs of physical mixtures (PMs) and hot-melt extrusion (HME) systems prepared with cocoa extract (CE) and the polymers eudragit E (EuE), plasdone S (PVP), and soluplus (Sol).

The morphological aspects of samples observed with optical microscopy and SEM (Figure 1) showed that CE and the polymers could be clearly distinguished in PM, in contrast to the HME samples, which appeared homogeneous. Moreover, SEM micrographs revealed the dense structure of HME, as expected [12].

**Figure 2:**
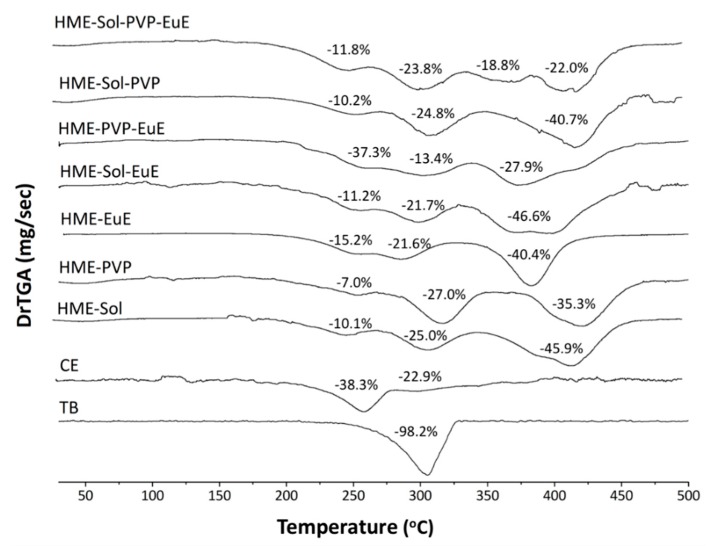
Thermogravimetric analyses (TGA) of the first derivative of solid dispersion systems produced by HME and containing EuE, PVP, and Sol in an inert atmosphere. The thermal profiles of theobromine (TB) and CE as supplied are also represented. Each weight loss event is indicated in figures as a percentage (%).

The thermal profile of mixtures was determined before (PM) and after the extrusion process (HME). TGA results showed no significant difference in thermal stability after processing. In fact, the extrudates presented a thermal profile equal to the sum of their individual compounds (Figure 2) without evidence of drug–polymer incompatibility.

**Figure 3:**
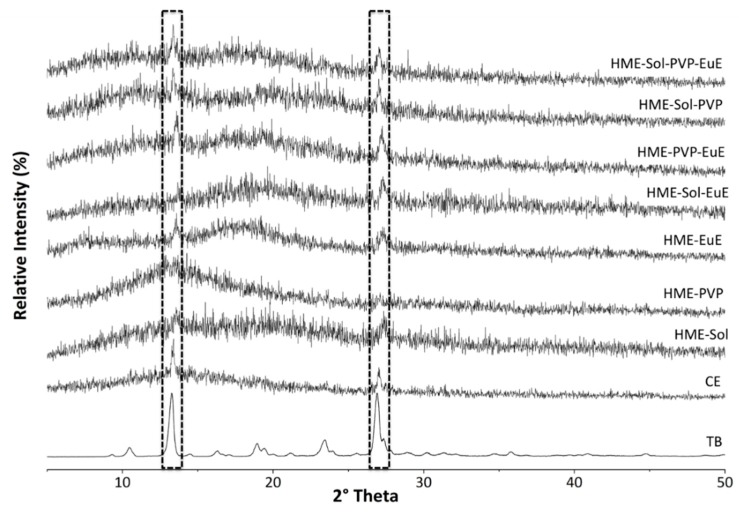
X-ray powder diffraction (XRPD) diffractograms of TB, CE, and HME systems. The peaks highlighted are characteristic of TB crystals.

The crystallinity of the systems could not be measured by thermal techniques once the TB melting event occurred after its thermal degradation [8]. Therefore, for this kind of verification, XRPD analyses were performed in both PM and HME samples, as well as in CE and TB as supplied. The main characteristic peaks of crystalline TB were also found in CE at 13.38° and 26.98° 2θ, together with an amorphous component (Figure 3).

XRPD of HME samples (Figure 3) indicated a strong amorphous component in all systems influenced by the polymers and the CE (both amorphous). Despite the dilution effect, peaks of the crystalline state of TB were able to be identified in almost all samples, reinforcing the stability of the crystalline lattice of this marker and its consequent incorporation into a pharmaceutical matrix.

**Figure 4:**
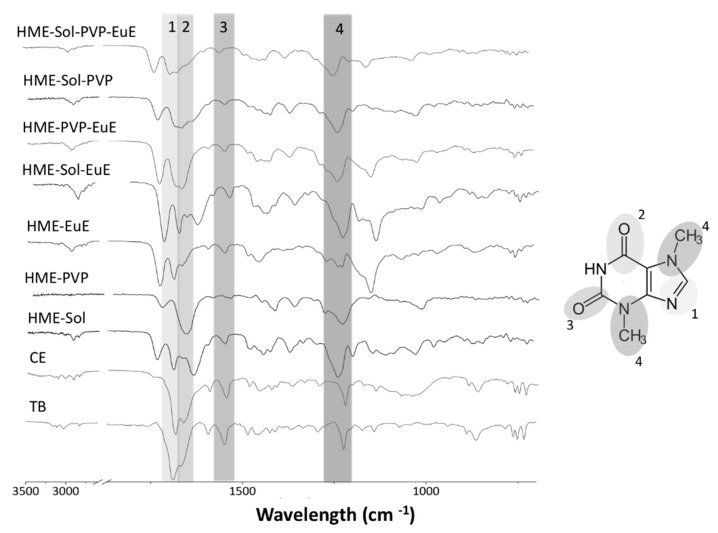
Fourier transform infrared spectroscopy (FTIR) spectra of TB, CE, and HME systems. The chemical structure of TB is shown, and their functional groups are shaded and numbered accordingly.

In FTIR spectra of CE, the main characteristic bands of TB were identified: in particular, the C=N stretching vibration band at 1689 cm^−1^, the band related to C–N at 1222 cm^−1^, and the bands associated with the two carbonyl group vibrations in the meta position at 1664 and 1544 cm^−1^ (Figure 4).

Nonetheless, small changes in the FTIR spectrum of HME samples mainly related to the intensity and position of some bands corresponding to the functional groups of TB suggest the interaction of this secondary metabolite with the polymeric matrix. Specifically, characteristic stretches with a slight shift or overlay were detected in HME-Sol, HME-Sol-EuE, and HME-Sol-EuE-PVP samples. In the HME PVP sample, C=N stretch and C–O were not visible. Moreover, the C=N stretches of HME Sol-PVP and PVP-EuE were not visible, reinforcing the probable drug–polymer interaction, possibly due to a higher degree of hydrogen bonding in the amorphous state [26].

### 3.3. Flow Evaluation

Flow behavior affects many industrial applications of pharmaceutical powders and is a crucial property in the development of oral solid dosage forms. Nevertheless, one of the main difficulties with bulk solid flow studies is the lack of reproducibility for most of the tests commonly used for this purpose, such as angle of repose and compressibility [27]. In this work, the assessment of multiple tests and the high degree of equipment automation allowed measures with a low variation coefficient (less than 3.5%), which resulted in a flowability index.

As expected, CE showed a low flowability (=49), proving its poor flow capacity (Table 2). Meanwhile, HME extrudates showed flowability in the range of 71–88, demonstrating a remarkable improvement of this property, which enables this processed natural product to be used in direct compression of tablets or to fill capsules, without any additional processing [28].

The thermal shear caused by HME is not only capable of greatly increasing the degree of interaction between the drug and polymer but also gives rise to a dense and uniform matrix producing granules with excellent flow characteristics, as previously described [9,11].

Together with the flowability determination, the dispersibility (%), which measures the tendency of a powder to scatter in the air, was also assessed. Once again, there was a great difference in performance between CE (37.8%) and HME extrudates (below 11%), as described in Table 2. A substantial improvement was achieved after HME processing, mitigating the possibility of air contamination in the production area.

**Figure 5:**
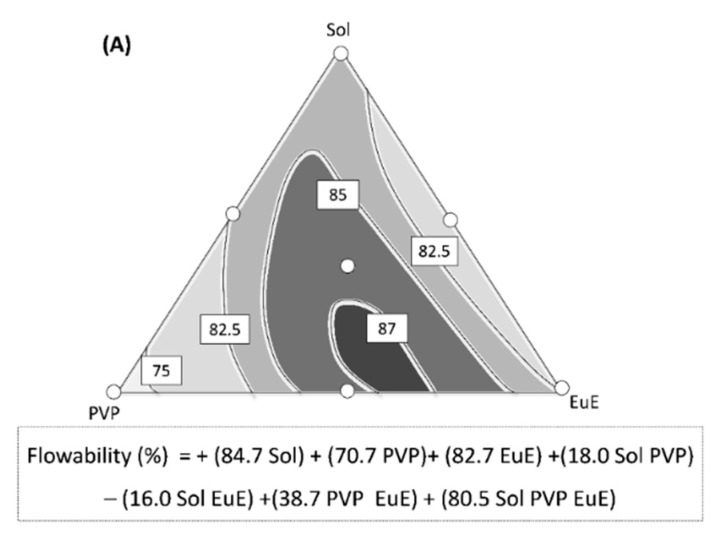

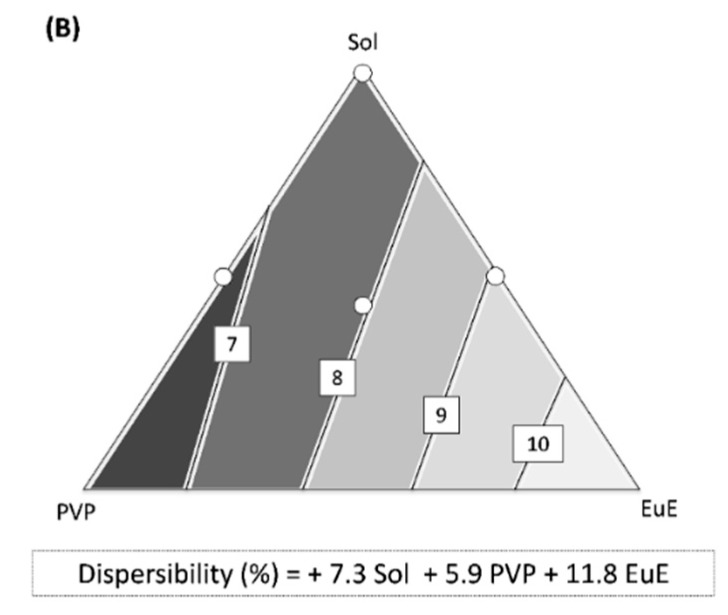
Contour diagrams for (**A**) flowability and (**B**) dispersibility together with their predictive equations. Dark areas show the regions with the best responses.

Although considerable improvements in flowability and dispersibility have been reached through HME processing, differences in these properties can be observed according to the composition of the polymer matrix, as shown in the response surfaces in Figure 5.

The predictive equation for flowability was adjusted to a special cubic model (*p* < 0.001) with *r*^2^ = 0.963 (Figure 5A). The three polymers used in this study, each contribute in equal terms to the flow improvement, showing high coefficient values with a positive signal (Figure 5A). The important contribution of the triple interaction between the polymers (high coefficient value) should also be highlighted, indicating a synergism among the different materials (Figure 5A).

The predictive equation for dispersibility was calculated using a linear method (*p* < 0.0001, *r*^2^ = 0.735; Figure 5B). In this case, there was no interaction between the formulation components. However, there are differences in polymer performance, especially for Sol and PVP, which may generate denser granules with lower potential for environmental contamination.

### 3.4. Wettability

**Figure 6:**
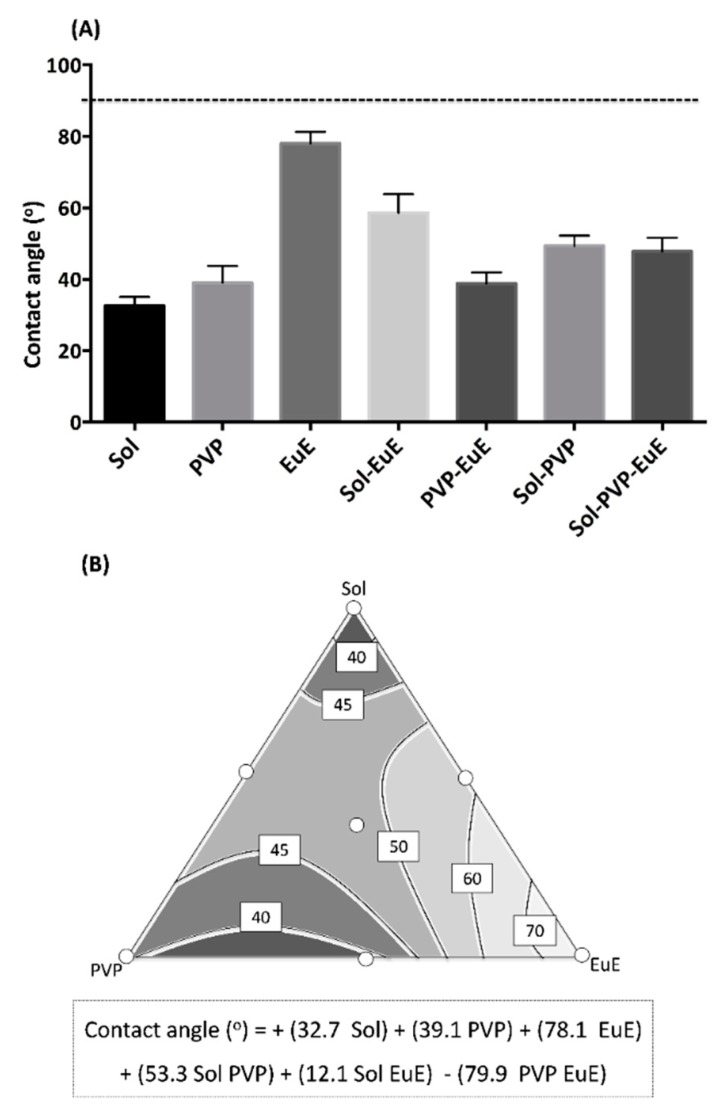
(**A**) Mean contact angle of HME systems. The dashed line indicates 90° (the limit to consider good wettability). (**B**) Contour diagram for the contact angle and predicted equation. Dark areas show the regions with the best responses.

Wettability is the ability of a solid surface to overcome the cohesive intermolecular interactions of water, mainly from hydrogen bonds, and to allow liquid spreading. This property is a sine qua non condition for the dissolution of solid dosage forms administered orally [29]. According to a previous report, CE presents a deficient wettability [7]. Figure 6A displays the contact angle results calculated for the seven HME extrudates. All samples presented a contact angle smaller than 90°, which indicates a favorable wetting of the extrudate surface. Similar results involving a hydrophilic HME matrix are described by other authors, due to the strong solid–liquid interaction achieved by these systems [30].

Although all formulations showed good wettability, marked differences could be observed between them, which indicate that the polymer composition plays an important role in this property. In fact, the response surface (Figure 6B) built from a quadratic model (*p* < 0.001; *r*^2^ = 0.940) reveals that Sol presents the lowest coefficient of the predictive equation, therefore leading to lower contact angles. Moreover, the combination of polymers favors the reduction of the contact angle, especially in the regions of the contour plot that combine PVP and EuE (significant interaction between these two polymers with a negative signal). The amphiphilic properties of Sol and the wettability enhancing activity attributed to PVP and EuE as described in the literature corroborate our results [31].

### 3.5. Dissolution Rate

**Figure 7:**
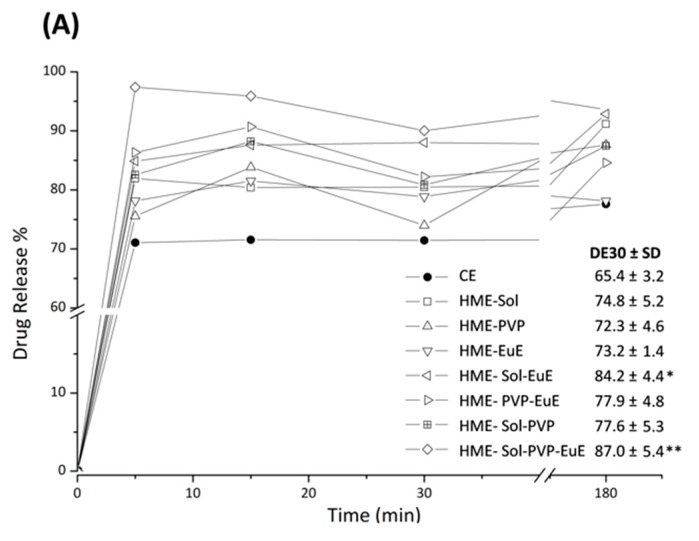

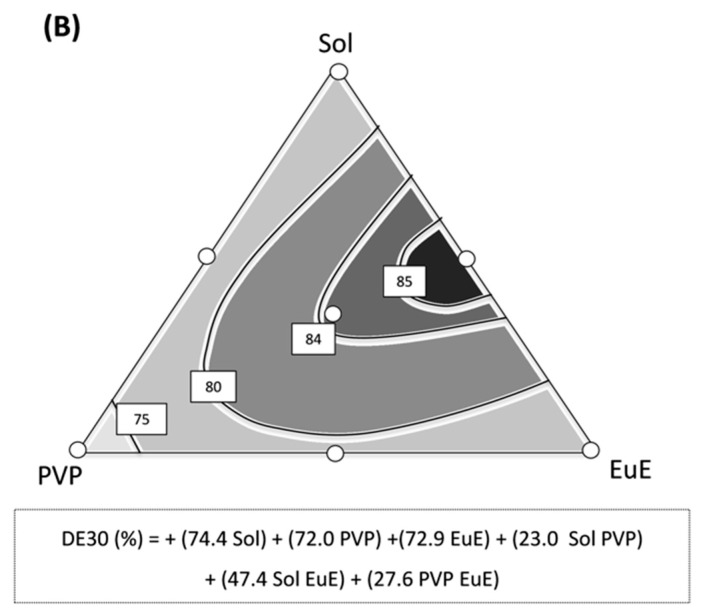
(**A**) Dissolution profiles of CE and HME systems together with the corresponding mean values of dissolution efficiency at 30 min (DE30) and the standard deviation (SD) in parentheses. Significant DE30 differences among samples are identified with * (*p* ≤ 0.01) and ** (*p* ≤ 0.0001). (**B**) Contour diagram for DE30 and predicted equation. Dark areas show the regions with the best responses.

Dissolution profiles of CE as supplied, compared with those of CE extrudates, are shown in Figure 7A. In contrast with the dissolution described for TB (CE marker), which shows a slow dissolution behavior [8], CE itself as well as HME formulations practically reached their maximum TB dissolution level in the initial minutes of the experiment. This behavior can be attributed to other components of the CE that might work as solubilizers. However, differences between the maximum solubilization levels were noticed between samples. While CE dissolves only 75% of the dose, the extrudates dissolved up to 95% of their doses, as in the case of HME Sol-EuE and HME Sol-EuE-PVP. Indeed, statistical analysis showed these two systems had better performance when compared with CE, with DE30 of 84 and 87%, respectively, against 65% as found for CE (Figure 7A).

As observed in other assays, the composition of the formulation has an important influence on the dissolution of HME samples. The quadratic model applied to this response (*p =* 0.011; *r*^2^ = 0.597) shows that regions composed of mixtures containing predominantly Sol and EuE led to DE30 results above 85%. In fact, the coefficient for the Sol-EuE interaction presented a high value, strongly contributing to the predictive equation (Figure 7B).

### 3.6. Prediction of the Optimized Formulation

**Figure 8:**
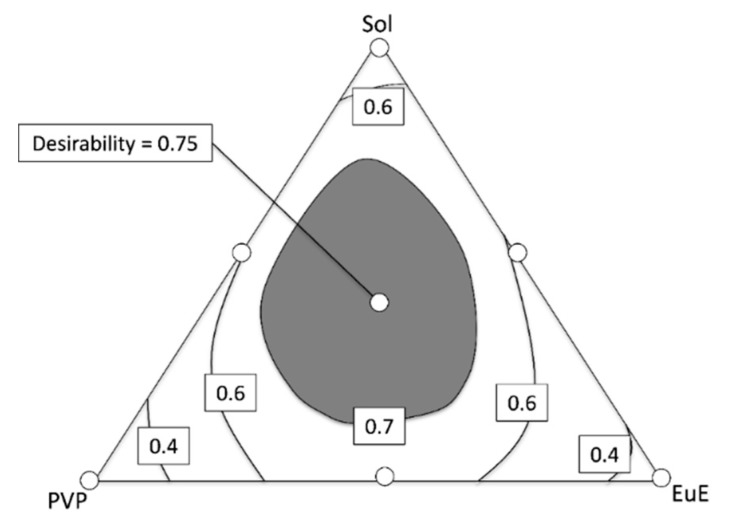
Contour diagrams for the optimized response considering flowability, dispersibility, contact angle, and DE30. Dark areas show the regions with higher desirability.

Mixture designs enable the determination, within the range of the excipient concentration studied, of regions in which different evaluated responses can be considered simultaneously [12]. In the case of this study, we chose to obtain an optimized response considering the four aspects of the study: DE30, flowability, contact angle, and dispersibility (Figure 8).

The highest desirability index (0.75) was found with a polymeric mixture composed of 23.1% Sol, 23.8% PVP, and 23.1% EuE, which practically corresponds to the central region of the contour plot. The expected responses for this formulation were DE30 of 84%, flowability of 87%, dispersibility of 8.4%, and contact angle of 48°, which are nearly the same as the results determined experimentally for the formulation HME Sol-PVP-EuE.

Moreover, a large central region, whose composition leads to desirability greater than 0.7, is observed in the contour plot (gray area of contour diagram). The possibilities with the best performance include a formulation containing 45.5% Sol, 11.9% PVP, and 12.6% EuE and a formulation containing 4.9% Sol, 32.2% PVP, and 33.6% EuE (Figure 8).

## 4. Conclusions

Solid dispersions of CE were effectively produced using a single-step HME process. Natural extract stability was preserved while dissolution and flow properties were improved. Despite the high stability of the TB crystal lattice, there was physicochemical evidence of drug-polymer interactions. The use of a mixture design allowed the identification of synergistic effects with excipient combination. In fact, the optimized formulation obtained considering four different responses showed that a mixture of Sol, PVP, and EuE in equal proportions produced the best results, making the pharmaceutical use of CE more feasible.

## Figures and Tables

**Table 1:** System composition and setup extrusion conditions, together with photomicrographs of the obtained filaments captured by optical microscopy. CE: cocoa extract; HME: hot-melt extrusion; Sol: Soluplus^®^; EuE: Eudragit E; PVP: Plasdone S.

Samples	CE % (*w*/*w*)	Polymer % (*w*/*w*)			Temperature (°C)	Rotation (rpm)	Drug Content (%)	Strip Aspect
		Sol	PVP	EuE				
**HME-Sol**	30	70	0	0	185	50	98.4 ± 0.3	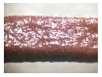
**HME-PVP**	30	0	70	0	170	100	101.5 ± 0.5	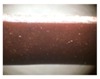
**HME-EuE**	30	0	0	70	150	100	103.7 ± 0.5	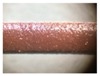
**HME-Sol-EuE**	30	35	35	0	160	100	90.1 ± 0.2	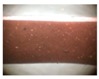
**HME-PVP-EuE**	30	0	35	35	160	100	94.1 ± 0.7	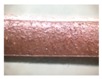
**HME-Sol-PVP**	30	35	35	0	170	100	99.0 ± 0.2	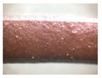
**HME-Sol-EuE-PVP**	30	23.3	23.3	23.3	150	100	98.3 ± 0.3	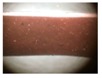

**Table 2:** Flow measurements of CE and HME systems. SD: standard deviation.

Sample	Repose Angle (° ± SD)		Spatula Angle (° ± SD)		Compressibility (%)	Flowability (%)	Dispersibility (%)
CE	53.4 ± 1.4	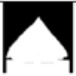	65.3 ± 1.5	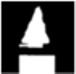	50.5 ± 1.7	49.0 ± 0.0	37.8 ± 3.2
HME Sol	31.5 ± 1.4	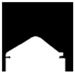	37.3 ± 1.1	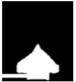	20.2 ± 1.7	85.5 ± 1.0	7.2 ± 0.6
HME PVP	42.1 ± 1.0	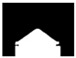	53.4 ± 1.4	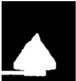	26.8 ± 0.9	71.5 ± 1.2	5.7 ± 0.3
HME EuE	34.7 ± 0.9	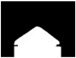	39.4 ± 1.0	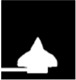	18.2 ± 0.0	83.5 ± 1.4	11.8 ± 2.7
HME PVP-Sol	33.6 ± 1.6	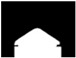	42.1 ± 2.6	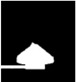	20.2 ± 1.7	81.5 ± 1.3	7.2 ± 0.2
HME PVP-EuE	33.6 ± 0.7	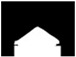	32.6 ± 1.0	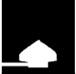	18.2 ± 2.6	86.0 ± 1.3	9.2 ± 1.0
HME Sol-EuE	35.0 ± 0.5	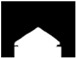	34.7 ± 0.3	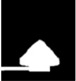	18.2 ± 0.0	84.0 ± 0.6	9.9 ± 1.0
HME EuE-Sol-PVP	32.6 ± 1.0	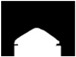	32.3 ± 1.2	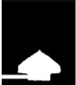	15.9 ± 3.3	88.0 ± 1.6	7.5 ± 1.0
